# Questing tick abundance in urban and peri-urban parks in the French city of Lyon

**DOI:** 10.1186/s13071-020-04451-1

**Published:** 2020-11-12

**Authors:** Laure Mathews-Martin, Manon Namèche, Gwenaël Vourc’h, Sabrina Gasser, Isabelle Lebert, Valérie Poux, Séverine Barry, Séverine Bord, Jeremy Jachacz, Karine Chalvet-Monfray, Gilles Bourdoiseau, Sophie Pamies, Diana Sepúlveda, Sandrine Chambon-Rouvier, Magalie René-Martellet

**Affiliations:** 1 INRAE, VetAgro Sup, UMR EPIA, Université Clermont Auvergne, 63122 Saint-Genès Champanelle, France; 2 INRAE, Communication et médias, Service d’appui à la recherche, Site de Theix, Université Clermont Auvergne, 63122 Saint-Genès-Champanelle, France; 3 INRAE, VetAgro Sup, UMR EPIA, Université de Lyon, 69280 Marcy l’Etoile, France; 4 Direction - Écologie Urbaine de la Ville de Lyon, Lyon, France; 5 Direction du Patrimoine Végétal, Métropole de Lyon, Lyon, France; 6 AgroParisTech, INRAE, UMR MIA-Paris, Université Paris-Saclay, 75005 Paris, France

**Keywords:** *Ixodes*, *Dermacentor*, Ticks, Park, Lyon, France

## Abstract

**Background:**

In Europe, ticks are responsible for the transmission of several pathogens of medical importance, including bacteria of the *Borrelia burgdorferi* (*s.l*.) complex, the agents of Lyme borreliosis. In France, the Auvergne Rhône-Alpes region is considered a hot spot for human tick-borne pathogen infections, with an estimated annual rate of 156 cases of Lyme borreliosis per 100,000 inhabitants. Although several studies have assessed the abundance of ticks in rural areas, little consideration has been given thus far to urban green spaces in France.

**Methods:**

This study aimed to estimate tick abundance in three parks, two urban (U1, U2) and one peri-urban (PU), in and around the city of Lyon (France). A forest in a rural area was used as a control (C). Tick sampling campaigns were performed in each site in April, May, June, July, and October 2019 using the dragging method. One hundred transects of 10 m^2^ each were randomly chosen in each park in places frequented by humans. The sampling sessions were carried out under semi-controlled abiotic conditions. Ticks were stored in 70% ethanol and identified to species and developmental stage under a light microscope using morphological keys.

**Results:**

A total of seven ticks (nymphs and adults) were collected in the two urban parks (six in U1 and one in U2), while 499 ticks were sampled in the peri-urban park. Of the 506 ticks collected, 504 were identified as *Ixodes ricinus*, one as *Dermacentor marginatus*, and one as *Ixodes frontalis*. In the peri-urban park, ticks were mainly collected under the forest cover and at forest edges. Tick density under forest cover was 7.1 times higher in the control site than in the peri-urban park throughout the survey period.

**Conclusions:**

This study confirmed the presence of ticks in all of the parks surveyed, although their occurrence in the urban parks was very rare compared to the peri-urban park and the control site. These results should serve as a basis for the implementation of preventive measures.
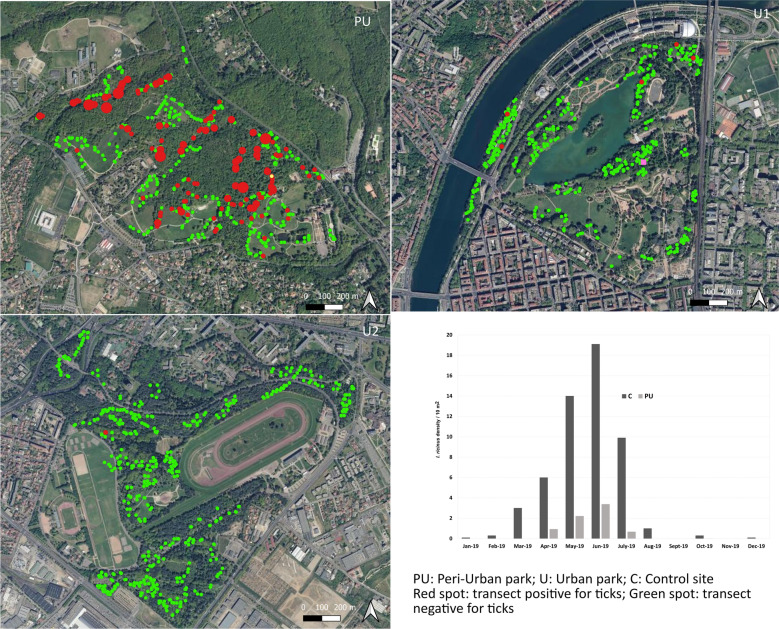

## Background

Ticks are the most important vectors of pathogens to humans and animals in Europe, especially in France. In particular, *Ixodes ricinus*, which is widespread in mainland France, is responsible for transmission of the agents of Lyme borreliosis, one of the most prevalent tick-borne zoonoses in Europe. A significant increase in human cases of Lyme borreliosis was noted in mainland France from 2015 to 2018, reaching 104 cases/100,000 inhabitants in 2018. The Auvergne-Rhône-Alpes region is one of the three most affected regions, with an estimated annual rate of 156 cases/100,000 inhabitants in 2016 [1, 2]*.* Although the risk of exposure to ticks, in particular to *I. ricinus*, has been previously assessed in several rural and peri-urban areas in France [3–6], urban parks have only rarely been explored [7–10].

Mitigating the risk of human exposure to ticks and tick-borne diseases has been a priority for the French public health authorities since the launch of the National Lyme Disease Plan in 2016 [11]. At a local scale, the implementation of this plan relies on the involvement of territorial health authorities who are responsible for the management of urban areas. However, these efforts could potentially be complicated by ‘re-greening’ initiatives in many cities. These actions aim to increase vegetated areas to improve the welfare of residents and enhance biodiversity levels. Such programmes could have the unintended side effect of expanding microhabitats suitable for tick development. Moreover, urban green spaces are highly frequented by humans. Thus, high tick densities in urban parks could lead to a high risk of human infection by tick-borne pathogens.

The aim of this study was to provide a preliminary snapshot of questing tick abundance and diversity in urban and peri-urban parks in the city of Lyon (France) using the dragging method of sampling. Our results were compared to a control site situated in a natural environment in the same area. In each park, we paid special attention to environments with a higher risk of tick exposure in order to provide useful data for the implementation of preventive measures.

## Methods

### Study sites

**Fig. 1.**
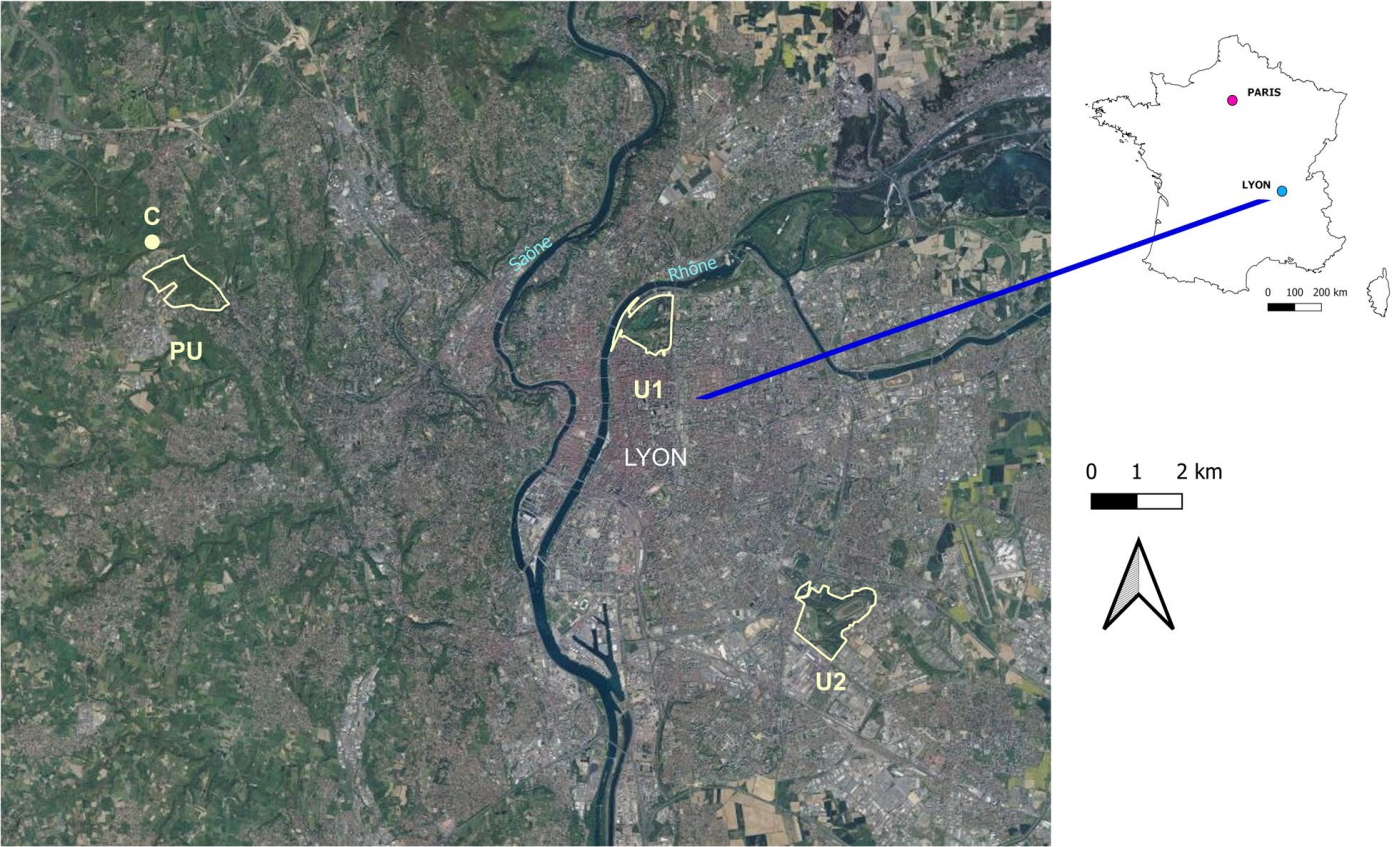
Location of the parks (U1, U2, PU) and control site (C) in the region of Lyon. Map background BDOrtho^®^ 50 cm IGN. *Abbreviations:* U1, U2, urban parks; PU, peri-urban park; C, control site

The survey was performed in three parks (two urban and one peri-urban) in and around the city of Lyon. The location of the study sites is presented in Fig. 1. With a population of 1,385,000 inhabitants, as estimated in 2017, Lyon and its outskirts represent the second-most populous metropolitan area in France (https://insee.fr). The city is equidistant between the northern Alps and the Monts d’Auvergne, at an altitude of 162–305 m above sea level. The eastern part of the city is an agricultural and industrial region with little relief. The western part (also called the Monts du Lyonnais area) is semi-mountainous and covered by forest. The closest peaks are located 15–20 km from the city center and culminate at 1004 meters (BD Alti^®^, IGN-F, https://www.ign.fr/).

Two of the three parks are within urban limits: Parc de la Tête d’Or-Brétillod (U1) is located in the center of the city of Lyon along the Rhône river, and Parc de Parilly (U2) is towards the south-east. The third, Parc de Lacroix-Laval (PU), is situated in a north-western suburban area (11 km from the center of Lyon) at the interface of the city and the foothills of the Monts du Lyonnais. Information on flora and fauna composition was obtained from the management staff of each park.

U1 is composed of a recreational park of 105 ha (Parc de la Tête d’Or) and an area of 7 ha (Parc du Brétillod). The site contains a 16-ha lake. Vegetation cover is mainly lawn with small groups of trees and a wooded area of approximately 6.5 ha. More than 8800 trees have been inventoried, with 61% of those being deciduous and 36.5% coniferous. Small areas are left unmanaged to limit the impact of mowing and tree cutting on biodiversity. The Brétillod area is subjected to flooding along the River Rhône. The majority of the trees in this area are deciduous. It also includes a meadow cut once a year which was explored as part of the study. Only domestic animals (cats, dogs, and horses) and small wild animals (e.g. birds and squirrels) are generally reported in U1.

U2 is a recreational park of 188 ha, which is considered a ‘green lung’ in the highly urbanized environment of eastern Lyon. More than 75% of the park is covered by grass, which is frequently cut, with small groups of trees (mainly coniferous). Only 2% of the park is managed as a semi-natural environment, with deciduous trees and brambles on the ground. Two small ponds are present. The animal community is roughly the same as in U1.

PU is a partially enclosed recreational park of 115 ha. Approximately 70% of the park is covered by woods, composed mainly of deciduous trees (90%), of which 80% are oaks and hornbeams. The open areas are meadows that are either mowed on a regular basis or only once or twice a year to meet an Ecolabel standard. A “jardin à la française” (French formal garden) is also accessible. A stream runs through the forest and two small ponds are present. The park is highly frequented by domestic and wild animals, including wild ungulates such as roe deer.

The 4-ha control site (C) has been monitored for questing ticks on a monthly basis since 2016 as part of the CCEID-CLIMATICK project [12]. The C site is characterized by wooded areas, forest edges, and roadside tracks. It is located 1 km away from the PU site, to which it is linked by an ecological corridor. Records collected in 2019 in the C site were used as the basis for comparison with the observations obtained for this study from the urban and peri-urban parks.

### Study design

In the three parks, surveillance campaigns were performed monthly, a few days apart, during the main period of tick activity, which is well-known thanks to the active surveillance that has been carried out in the region since 2016 as part of the CCEID-CLIMATICK project.

In order to collect as many ticks as possible while maximizing the efficiency of collection, we selected days for sampling that had optimal meteorological conditions for the activity of ticks, *I. ricinus* in particular. Specifically, the days of sampling were chosen using the following criteria: rainless, windspeed less than 10 km/h, temperature under the forest cover between 12–25 °C, and humidity under the forest cover at or above 50%. For this, a weather station was placed 20 cm from the ground under tree cover in each park to monitor local temperature and hygrometry (HOBO^®^, MX2300, MX2302, Onset Computer Corporation^®^). The time period during the day for the collection of questing ticks was determined using the temperature and hygrometry curves of the five previous days and systematically confirmed by temperature and hygrometry records at the beginning and the end of the collection.

Every month, 100 different transects of 10 m^2^ were randomly selected in each park. Transects were not the same from month to month. The aim was to explore all environments that could be frequented by humans. In the control site, the same 10 transects of 10 m^2^ were sampled every month.

Five types of vegetation were defined: forest, footpath/track inside the forest, forest edge, footpath/track in an open area, and meadow. Forest and footpath/track inside the forest were described as closed environments (CE), forest edge as a transitional environment (TE), and footpath/track in an open area and meadow as open environments (OE). A GPS waypoint (Garmin, Dakota^®^ 10) and a picture were taken at each transect.

### Tick collection, storage, and identification

Ticks were collected using a 1-m^2^ white flannel cloth that was dragged on the ground [13]. Both sides of the cloth were closely examined for the presence of ticks. Only nymphs and adults were collected. All questing ticks were stored in Eppendorf tubes in 70% ethanol at ambient temperature until morphological identification was performed under a light microscope using identification keys [14].

### Data analysis

Positive transects (transects where at least one tick was collected) and tick densities (number of ticks per sampled area of 10 m^2^) were mapped using QGIS software (Desktop 3.4.3 [15]). A Chi-square Test or Fisher’s exact test was performed to compare the number of tick-positive transects between parks or between environments within a park. For all tests, the significance threshold was defined as *P* < 0.05. The statistical analyses were performed using R (4.0.0.) [16].

## Results

### Tick collection in parks

In the three parks, surveillance campaigns were performed monthly, a few days apart, in April, May, June, July, and October 2019, when meteorological conditions fulfilled the criteria of the study design. No sampling was performed in August and September due to the particularly hot and dry weather conditions.

**Fig. 2.**
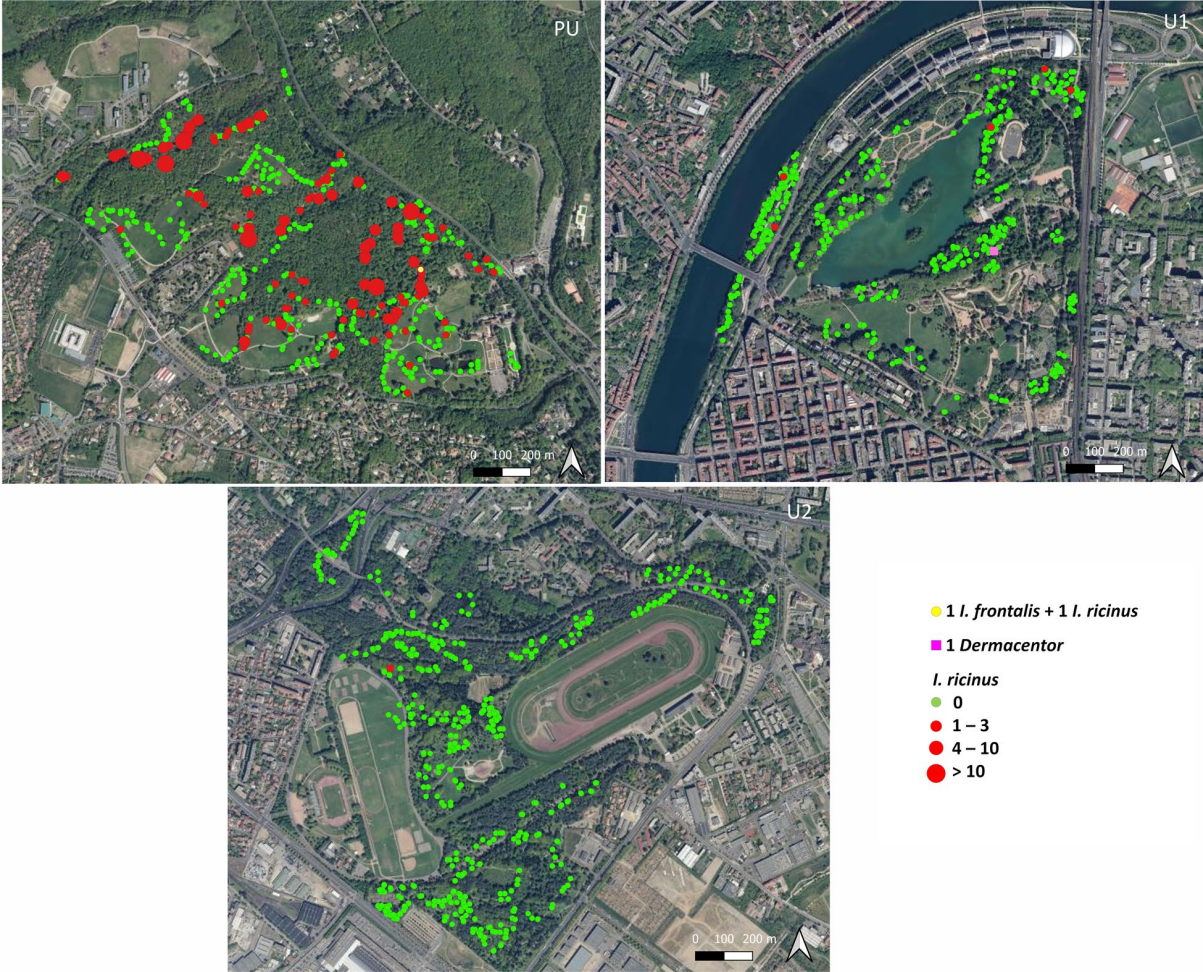
Distribution of ticks in PU, U1, and U2 sites. *Key*: red spot, transect positive for *I. ricinus* ticks; green spot, transect negative for *I. ricinus* ticks. Tick number is expressed per 10-m^2^ transect. Map background BDOrtho^®^ 50 cm IGN. *Abbreviations*: U1, U2, urban parks; PU, peri-urban park

A total of 506 ticks were collected (499 in PU, 6 in U1, 1 in U2) from the 5000 m^2^ area (100 × 10 m^2^ × 5 visits) surveyed in each park during the study period. The majority of ticks were identified as *I. ricinus* (*n* = 504, of which 94% (*n* = 474) were nymphs and 6% (*n* = 30) were adults). We also identified one adult male of *Dermacentor marginatus* (in U1) and one nymph of *Ixodes frontalis* (in PU). The spatial distribution of tick-positive and -negative transects in the three parks surveyed, and their relative tick densities, are presented in Fig. 2. Tick densities observed in the peri-urban park (PU) were at least 100 times higher than those observed in the urban parks (U1, U2) (Table 1). Specifically, 29.2% (146/500 transects) of the transects yielded ticks in PU versus 1.2% in U1 (6/500 transects) and 0.2% in U2 (1/500 transects). The number of positive transects was significantly different among the parks (Chi-square test, *χ*^2^ = 464.85, *df* = 3, *P* < 0.0001).

**Table 1.** Density of questing ticks and percentage of positive transects in the three parks throughout the study period

Site	PU	U1	U2
Tick number/area (m^2^)	499/5000	6/5000	1/5000
Density/10 m^2^	1.00	0.01	0.002
Positive transects/total transects	146/500	6/500	1/500
Percentage	29.2	1.2	0.2

*Notes*: Tick number/area (m^2^): total number of ticks/sampled area in m^2^; positive transects/total transects: number of tick-positive transects/total number of transects

### Tick densities in closed, transitional, and open environments in PU

Of the three parks surveyed, only the PU site contained all three distinct types of environments (CE, TE, and OE) and an adequate sample size of questing ticks from each type to permit an analysis of the habitat effect (Table 2). Tick density (corresponding to 498 *Ixodes ricinus* and 1 *Ixodes frontalis* in PU) was estimated to be 32 times higher in closed environments (CE) and 16 times higher in transitional environments (TE) than in open environments (OE). Similarly, a higher number of positive transects was found in CE (44.4%; 119/268 transects) than in TE (22.4%; 19/85 transects) or OE (5.4%; 8/147 transects). Throughout the study period, there was a significant difference among the environment types in PU concerning the number of positive transects (Chi-square test, *χ*^2^ = 72.02, *df* = 2, *P* < 0.0001).

**Table 2.** Density of questing ticks and percentage of positive transects in the three environments in the periurban (PU) location throughout the study period

Environment type	CE	TE	OE
Tick number/area (m^2^)	425/2680	65/850	8/1470
Density/10 m^2^	1.6	0.8	0.05
Positive transects/total transects	119/268	19/85	8/147
Percentage	44.4%	22.4%	5.4%

*Notes*: Tick number/area (m^2^): total number of ticks/sampled area in m^2^; positive transects/total transects: number of tick-positive transects/total number of transects

*Abbreviations*: CE, closed environment, within forests and along footpaths or tracks in forests; TE, transitional environment, forest edge; OE, open environment, meadow or along footpaths or tracks in open areas

### Comparison of PU with the control site (C)

**Fig. 3.**
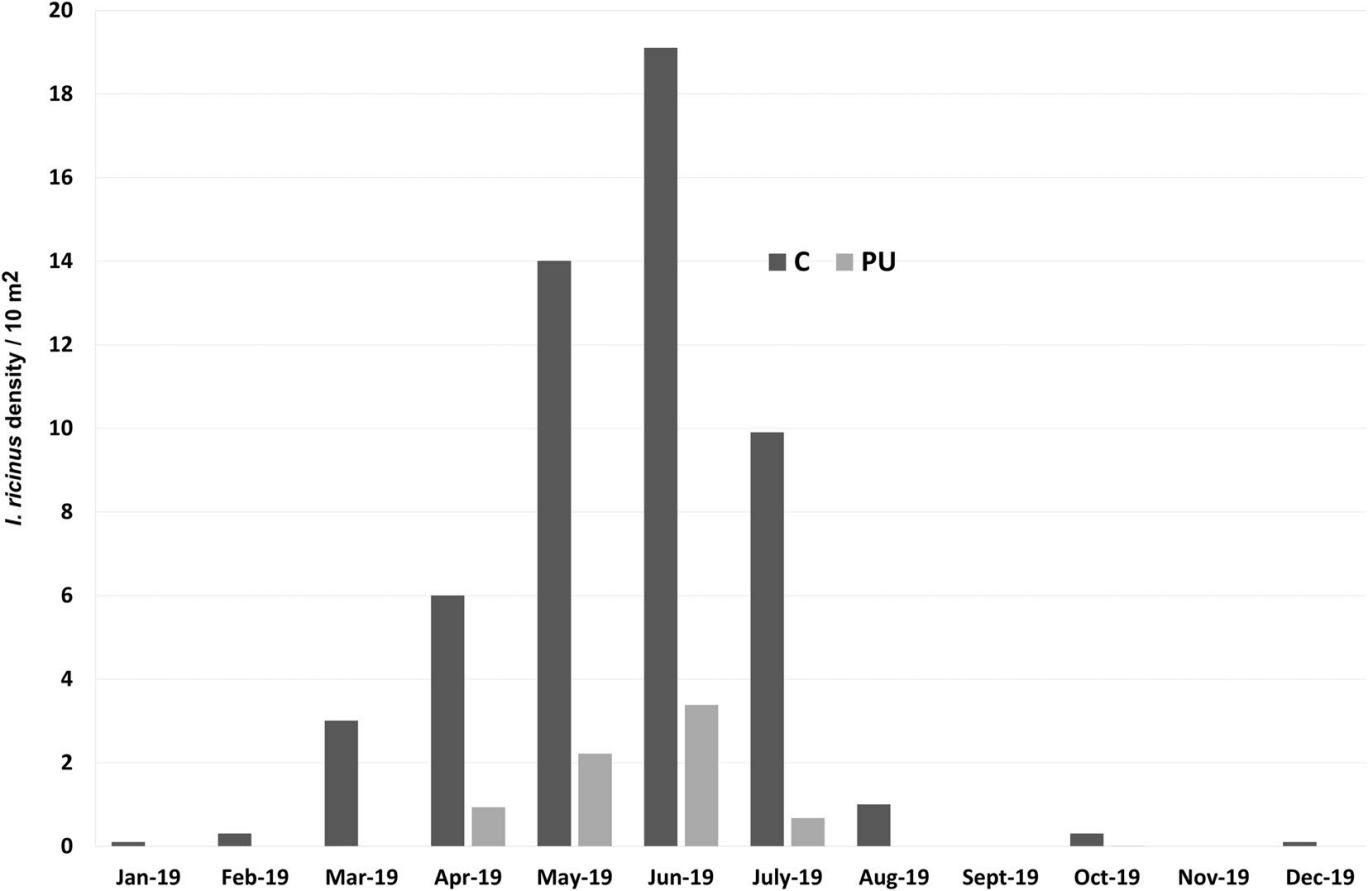
Seasonality of *Ixodes* spp. activity in PU park and C site. 1000 m^2^ in PU and 100 m^2^ in C were sampled each month. *Abbreviations*: PU, peri-urban park; C, control site

A total of 493 ticks (all identified as *I. ricinus*) were collected in April, May, June, July, and October (10 transects per month) in the 500 m^2^ area (10 × 10 m^2^ × 5 visits) sampled at site C. Of the questing ticks collected, 98% (*n* = 483) were nymphs and 2% (*n* = 10) were adults. Only closed and transitional environments were present in this location. We thus compared tick densities and the number of positive transects between the PU and C locations, considering only ticks that were sampled in closed and transitional environments (Table 3). The highest tick density was observed in June in both sites (Fig. 3). Due to the low number of questing ticks collected in the urban parks (U1 and U2), these sites were excluded from comparisons to site C. In these parks, it was not possible to identify any seasonal pattern of tick activity. Tick density was higher in site C than in PU, regardless of the period assessed (all five months or only June) (Table 3). During the peak of activity in June, tick density was 5.5 times higher in site C (19.1 ticks/10 m^2^) than in PU (3.5 ticks/10 m^2^). Overall, tick density was 7.1 times higher in site C than in PU.

**Table 3.** Density of questing ticks and percentage of positive transects in closed and transitional environments in periurban (PU) and control (C) sites

Time frame	Entire period		June	
Site	PU	C	PU	C
Tick number/area (m^2^)	490/3530	493/500	223/640	191/100
Density/10 m^2^	1.4	9.9	3.5	19.1
Positive transects/total transects	138/353	40/50	48/64	10/10
Percentage	39	80	75	100

*Notes*: Entire period: cumulative data for all five months; Tick number/area (m^2^): total number of ticks/sampled area in m^2^ in closed and transitional environments; positive transects/total transects: number of tick-positive transects/total number of transects in closed and transitional environments

When examining the overall percentage of positive transects from closed and transitional environments, we detected a statistically significant difference between the two study sites. Specifically, the percentage of positive transects was two times higher in site C than in PU (Fisherʼs exact test, *P* < 0.0001). However, such a difference was not observed in June (Fisherʼs exact test, *P* = 0.11).

## Discussion

The aim of the present study was to estimate tick abundances in urban and peri-urban parks in the city of Lyon (France). We confirmed the presence of ticks in all parks surveyed, mainly of *I. ricinus*, with significant differences in abundance among parks.

The earliest studies on the abundance of ticks in urban environments date back to the late 1980s in the USA [17]. Lyme disease is endemic in the USA, and this type of survey has been used in many published studies [18, 19]. Likewise, the presence of ticks in urban parks has been investigated in several European countries [10, 20–24]. In France, the first surveys of ticks in urban environments (specifically, in and around the city of Lyon) date back to the 1990s [9, 25], but have not been updated since. Recently, participatory tools have been developed to allow citizens to report tick bites. These declarations have indicated a high incidence of tick bites in urban and peri-urban areas. Specifically, 29% of tick bites reported *via* the “Signalement-Tique” application from July 2017 to December 2018 occurred, according to the declarants’ assertions, in parks and gardens [26]. Likewise, in Belgium, 38% of tick bites were recorded near the houses of the respondents, and 2.2% in a city park, respectively [27]. To further evaluate the risk of human-tick encounters, efforts have been made in Europe to develop active surveillance programmes of ticks in parks that are highly frequented by humans.

Temperature and relative humidity are known to shape the host-seeking activity of ticks [28, 29]. Thus, by using weather stations, we were able to choose days for collection that had appropriate abiotic conditions, in particular suitable temperature and humidity near the ground. In cities, abiotic factors are of primary importance for the survival of the off-host stages of ticks. The urban heat island effect has been linked to reduced tick abundance and activity, and therefore a lower prevalence of Lyme disease [21]. Previous tick abundance surveys performed in Lyon and its outskirts [9, 25] focused on a single sampling event (30 min) of 46 different sites in spring, in order to increase the coverage area. Here, instead, we chose to focus on only three parks, but with monthly surveillance and a higher number of samples in each park. The sampling effort targeted the areas that are most frequented by humans. Consequently, areas that were overgrown by vegetation or less accessible were not sampled. Throughout the five months of the survey, we explored 5000 m^2^ in each park (15,000 m^2^ in total), which represents 0.27% of the area of U2, 0.43% of PU, and 0.52% of U1. As a comparison, a total of 12,420 m^2^ were sampled in 15 urban and suburban plots in the outskirts of Antwerp, in Belgium, in 2014 and 2016. In that study, the sampling effort varied from 0.07% for the largest plot (132 ha) to 1.63% for a suburban plot of 10.3 ha [24]. In terms of abundance, the values we obtained in PU were higher than those recorded in Italy in 2012, where the highest density was 0.63 ticks per 10 m^2^ in a thickly forested natural reserve situated on the outskirts of Imola [20]. Tick density in PU was similar to that found in Belgium, where the abundance of *I. ricinus* in the largest suburban plot sampled (132 ha) was 1.5 ticks per 10 m^2^ (nymphs and adults). It is, however, difficult to compare abundances among countries due partly to climatic differences, which have also been shown to influence tick abundance and activity [28, 29].

The discrepancies regarding tick abundances between the three analyzed parks could be due to various factors. First, PU is regularly frequented by wild fauna, especially wild ungulates (confirmed by the observations of the park employees). Instead, these animals are extremely rarely observed in the intra-urban parks. Here, the low tick densities observed within the urban parks are likely the result of (i) altered ecological corridors that do not allow wild ungulates to easily enter these locations and (ii) the (probably) low contribution of other large animals (including domestic animals, which may be treated with acaricides on a regular basis) to the maintenance of tick life-cycles.

Another factor that likely contributed was the habitat composition of the sites. Of the three sites, PU has the most favorable habitat for *Ixodes* spp. ticks, with a forest cover that is mainly composed of hardwoods and brambles in the undergrowth [30]. The Parc du Brétillod (in U1) is a greenway that connects with the Parc de la Feyssine and Grand parc de Miribel-Jonage, which are located upstream on the right bank of the Rhône. This space is managed as naturally as possible. However, the soil is very sandy and does not retain much moisture, which probably limits tick establishment. The remainder of U1 (Parc de la Tête d’Or) and U2 have much sparser woodland cover and are regularly mowed, which is also unfavorable for tick presence.

As a control, we used a site on the north-western outskirt of the city of Lyon that has been surveyed for questing ticks since 2016; data from this site were used for comparison of both tick density and the seasonal peak of activity. During the surveillance period, similar patterns of *Ixodes* spp. activity were observed between the PU and C sites, but tick density was 7.1 times higher in C than in PU. These results suggest that tick abundance increases from an urban center to the outskirts, as previously reported in the same area [9, 25] and in recent studies performed in Europe [4, 20–22, 24, 31, 32]. In the 1990s, the north-eastern and western peri-urban areas of Lyon were highlighted as being more at risk with respect to tick abundance, given their proximity to the wooded mountainous region of Les Monts du Lyonnais [9].

In the Auvergne-Rhône-Alpes region, ticks from the genera *Dermacentor* and *Ixodes* are widespread; the former is responsible for transmission of the agents of canine and equine piroplasmosis, while the latter carries bacteria of the *Borrelia burgdorferi* (*s.l.*) complex, which cause Lyme disease [8]. Previous studies in and around the city of Lyon reported only ticks of the genus *Ixodes* [8, 9]. This pattern of tick species diversity was also observed in the present study, where *Ixodes* spp. ticks represented the vast majority of collections. The only exception was a single *Dermacentor marginatus* tick collected in U1. *Dermacentor* ticks are generally found in rural areas, in open environments such as grazed meadows, roadsides, and forest edges [33], and, at the adult stage, often parasitize horses and dogs. Immature stages are endophilic (nidicolous) and can be found on rodents. The U1 park often hosts horses that are bred in meadows outside of the city and is also highly frequented by dogs. *Ixodes frontalis*, a bird-associated tick [34], can also be occasionally found questing on vegetation [35]. However, there is to date no evidence of local (i.e. intra-urban) reproduction of either species.

Because this was a preliminary study, screening for potential tick-borne pathogens was not performed. Previous work has shown that the pathogen burden in ticks may be higher in developed urban areas compared to rural areas [21]. In a study conducted in this same region in the 1990s, 13.5% of the ticks analysed were infected by *Borrelia burgdorferi* (*s.l.*), reaching 14.5% in Les Monts du Lyonnais [25]. In Switzerland, the infection rate of urban ticks was shown to be equivalent to that of rural areas, with a tendency toward co-infection (bacteria, viruses, parasites) [23]. Thus, tick-borne pathogen surveillance should be a priority in the future to evaluate the risk of human exposure to tick-borne pathogens. Although the overall human threat in urban and peri-urban parks appears to be low, the risk, which is the combination of the hazard and human exposure, could still be high due to increased visitation rates by humans, and must be taken into account in the design of preventive measures.

## Conclusions

This study confirmed the presence of ticks, mainly of the species *I. ricinus*, in all three parks surveyed, although notable differences in tick density were observed. Such differences are probably linked with variations in landscape connectivity and the densities of wild fauna, especially ungulates. Notably, ticks were rarely found in intra-urban parks, particularly when compared to a peri-urban environment in the same period. Tick density was much higher in forests and forest edges than in lawns, where the number of ticks was low. A more-accurate description of green-space management, human activities, and a fauna inventory could help to improve our understanding of the factors that influence tick densities and tick bite risk within urban parks. Surveillance programs should be continued to assess the possible introduction of new tick species, particularly in the contexts of global warming and of urban policies of ‘re-greening’.

## Data Availability

The datasets used and/or analysed in the present study are available from the corresponding author upon reasonable request.

## Abbreviations

U1: Urban park 1 (Tête-d’Or and Brétillod, Lyon, France)
U2: Urban park 2 (Parilly, Bron, France)
PU: Peri-urban park (Lacroix-Laval, Charbonnières-les-Bains, France)
C: Control site (La Tour de Salvagny, France)
GIS: Geographical information system

## References

[1] Vaissière E, Thabuis A, Couturier E (2018). Surveillance de la borréliose de Lyme.

[2] Fournier L, Roussel V, Couturier E, Jaulhac B, Goronflot T, Septfons A. Épidémiologie de la borréliose de Lyme en médecine générale, France métropolitaine, 2009–2016. BEH; 2018. p. 19–20.

[3] Paul RE, Cote M, Le Naour E, Bonnet SI (2016). Environmental factors influencing tick densities over seven years in a French suburban forest. Parasites Vectors.

[4] Marchant A, Le Coupanec A, Joly C, Perthame E, Sertour N, Garnier M (2017). Infection of *Ixodes ricinus* by *Borrelia burgdorferi* sensu lato in peri-urban forests of France. PLoS ONE.

[5] Papillon P, Dodier R (2011). Periurban forests shifting from recreation to wellness. J Alp Res.

[6] Dernat S, Johany F (2019). Tick bite risk as a socio-spatial representation - an exploratory study in Massif Central, France. Land.

[7] Dutraive J (2019). Etude de la corrélation entre la densité de la tique *Ixodes ricinus* et la densité des chevreuils en région toulousaine.

[8] Gilot B, Perez-Eid C (1998). Bio-écologie des tiques induisant les pathologies les plus importantes en France. Med Mal Infect..

[9] Pichot J, Gilot B, Almire N, Polette K, Degeilh B (1997). *Ixodes* populations (*xodes ricinus* Linné, 1758; *Ixodes hexagonus* Leach, 1815) in the city of Lyon (France) and its outskirts. Parasite.

[10] Rizzoli A, Silaghi C, Obiegala A, Rudolf I, Hubalek Z, Foldvari G (2014). *Ixodes ricinus* and its transmitted pathogens in urban and peri-urban areas in Europe: new hazards and relevance for public health. Front Public Health.

[11] Ministère des Solidarités et de la Santé. Plan national de lutte contre la maladie de Lyme et les maladies transmissibles par les tiques. 2016;27. https://solidarites-sante.gouv.fr/IMG/pdf/plan_lyme_180117.pdf.

[12] Chalvet-Monfray K. Bilan de 5 ans de suivi mensuel d’un réseau d’observatoires d’*Ixodes ricinus*. In: Réunion Tiques & Maladies à Tiques, 22–23 May 2019, Montpellier, France.

[13] Boyard C, Barnouin J, Gasqui P, Vourc'h G (2007). Local environmental factors characterizing *Ixodes ricinus* nymph abundance in grazed permanent pastures for cattle. Parasitology.

[14] Estrada-Peña A, Mihalca A, Petney T (2017). Ticks of Europe and North Africa: a guide to species identification.

[15] QGIS Development Team. QGIS Geographic Information System. 2018. https://qgis.org/.

[16] R Development Core Team. R: a language and environment for statistical computing. 2020. https://www.R-project.org/.

[17] Falco RC, Fish D (1989). Potential for exposure to tick bites in recreational parks in a Lyme disease endemic area. Am J Public Health.

[18] Noden BH, Loss SR, Maichak C, Williams F (2017). Risk of encountering ticks and tick-borne pathogens in a rapidly growing metropolitan area in the US Great Plains. Ticks Tick Borne Dis.

[19] Mead P, Hook S, Niesobecki S, Ray J, Meek J, Delorey M (2018). Risk factors for tick exposure in suburban settings in the northeastern United States. Ticks Tick Borne Dis.

[20] Corrain R, Drigo M, Fenati M, Menandro ML, Mondin A, Pasotto D (2012). Study on ticks and tick-borne zoonoses in public parks in Italy. Zoonoses Public Health.

[21] Buczek A, Ciura D, Bartosik K, Zając Z, Kulisz J (2014). Threat of attacks of *Ixodes ricinus* ticks (*Ixodida: Ixodidae*) and Lyme borreliosis within urban heat islands in south-western Poland. Parasites Vectors.

[22] Nelson C, Banks S, Jeffries CL, Walker T, Logan JG (2015). Tick abundances in south London parks and the potential risk for Lyme borreliosis to the general public. Med Vet Entomol.

[23] Oechslin CP, Heutschi D, Lenz N, Tischhauser W, Péter O, Rais O (2017). Prevalence of tick-borne pathogens in questing *Ixodes ricinus* ticks in urban and suburban areas of Switzerland. Parasites Vectors.

[24] Heylen D, Lasters R, Adriaensen F, Fonville M, Sprong H, Matthysen E (2019). Ticks and tick-borne diseases in the city : role of landscape connectivity and green space characteristics in a metropolitan area. Sci Total Environ.

[25] Quessada T, Martial-Convert F, Arnaud S, Heudet de la Vallée H, Gilot B, Pichot J (2003). Prevalence of *Borrelia burgdorferi* species and identification of *Borrelia valaisiana* in questing *Ixodes ricinus* in the Lyon region of France as determined by polymerase chain reaction-restriction fragment length polymorphism. Eur J Clin Microbiol Infect Dis.

[26] Durand J, Brun-Jacob A, Gallon C, Carravieri I, Marchand J, Palin B, et al. CiTIQUE, les sciences participatives au service de tous. In: Réunion Tiques & Maladies à Tiques, 22–23 May 2019, Montpellier, France.

[27] Tersago K, Leroy M, Lernout T. TiquesNet. Surveillance des morsures de tiques en Belgique. 2018. https://www.sciensano.be/fr/projets/tiquesnet.

[28] Ruiz-Fons F, Acevedo P, Gortázar C, de la Fuente J (2012). Factors driving the abundance of *Ixodes ricinus* ticks and the prevalence of zoonotic *I. ricinus*-borne pathogens in natural foci. Appl Environ Microbiol.

[29] Agoulon A, Butet A, Hoch T, Perez G, Plantard O, Verheyden H, Karen DM, Nathalie B (2016). 3. Dynamique des populations de tiques et liaison avec les facteurs environnementaux. Tiques et maladies à tiques: biologie, écologie évolutive, épidémiologie.

[30] Tack W, Madder M, Baeten L, De Frenne P, Verheyen K (2012). The abundance of *Ixodes ricinus* ticks depends on tree species composition and shrub cover. Parasitology.

[31] Hansford KM, Fonville M, Gillingham EL, Coipan EC, Pietzsch ME, Krawczyk AI (2017). Ticks and *Borrelia* in urban and peri-urban green space habitats in a city in southern England. Ticks Tick Borne Dis.

[32] Pangrácová L, Derdáková M, Pekárik L, Hviščová I, Víchová B, Stanko M (2013). *Ixodes ricinus* abundance and its infection with the tick-borne pathogens in urban and suburban areas of eastern Slovakia. Parasites Vectors.

[33] Nosek J (1972). The ecology and public health importance of *Dermacentor marginatus* and *D. reticulatus* ticks in central Europe. Folia Parasitol.

[34] Pérez-Eid C. Les tiques: identification, biologie, importance médicale et vétérinaire. Editions Tec et doc et Editions médicales internationales edn. Paris et Cachan, France: Lavoisier; 2007.

[35] Agoulon A, Hoch T, Heylen D, Chalvet-Monfray K, Plantard O (2019). Unravelling the phenology of *Ixodes frontalis*, a common but understudied tick species in Europe. Ticks Tick Borne Dis.

